# CCL3–CCR5 axis contributes to progression of esophageal squamous cell carcinoma by promoting cell migration and invasion via Akt and ERK pathways

**DOI:** 10.1038/s41374-020-0441-4

**Published:** 2020-05-26

**Authors:** Takayuki Kodama, Yu-ichiro Koma, Noriaki Arai, Aya Kido, Naoki Urakawa, Mari Nishio, Manabu Shigeoka, Hiroshi Yokozaki

**Affiliations:** 1 Division of Pathology, Department of Pathology, Kobe University Graduate School of Medicine, Kobe, Japan; 2 Department of Molecular Pathology, Institute of Biomedical and Health Sciences, Hiroshima University, Hiroshima, Japan; 3 Division of Gastro-intestinal Surgery, Department of Surgery, Kobe University Graduate School of Medicine, Kobe, Japan

**Keywords:** Cancer microenvironment, Oesophageal cancer, Oncogenesis

## Abstract

Tumor-associated macrophages (TAMs) contribute to the progression and mortality of various malignancies. We reported that high numbers of infiltrating TAMs were significantly associated with tumor progression and poor prognosis in esophageal squamous cell carcinoma (ESCC). In our previous investigation of TAMs’ actions in ESCC, we compared gene expression profiles between peripheral blood monocyte (PBMo)-derived macrophages and TAM-like macrophages stimulated with conditioned media of ESCC cell lines. Among the upregulated genes in the TAM-like macrophages, we focused on *CC chemokine ligand 3* (*CCL3*), which was reported to contribute to tumor progression in several malignancies. Herein, we observed that not only TAMs but also ESCC cell lines expressed CCL3. A CCL3 receptor, CC chemokine receptor 5 (CCR5) was expressed in the ESCC cell lines. Treating the ESCC cell lines with recombinant human (rh)CCL3 induced the phosphorylations of Akt and ERK, which were suppressed by CCR5 knockdown. Migration and invasion of ESCC cells were promoted by treatment with rhCCL3 and co-culture with TAMs. TAMs/rhCCL3-promoted cell migration and invasion were suppressed by inhibition of the CCL3–CCR5 axis, PI3K/Akt, and MEK/ERK pathways. Treatment with rhCCL3 upregulated *MMP2* and *VEGFA* expressions in ESCC cell lines. Our immunohistochemical analysis of 68 resected ESCC cases showed that high expression of CCL3 and/or CCR5 in ESCC tissues was associated with poor prognosis. High CCR5 expression was associated with deeper invasion, presence of vascular invasion, higher pathological stage, higher numbers of infiltrating CD204^+^ TAMs, and higher microvascular density. High expression of both CCL3 and CCR5 was an independent prognostic factor for disease-free survival. These results suggest that CCL3 derived from both TAMs and cancer cells contributes to the progression and poor prognosis of ESCC by promoting cell migration and invasion via the binding of CCR5 and the phosphorylations of Akt and ERK. The CCL3–CCR5 axis could become the target of new therapies against ESCC.

## Abstract (web summary)

The authors. show that CCL3 derived from both tumor-associated macrophages and esophageal squamous cell carcinoma (ESCC) cells promotes cell migration and invasion of ESCC cells via binding CCR5. High expression of CCL3 and/or CCR5 associates with poor prognosis in ESCC patients. CCL3–CCR5 axis could be a specific target of anti-cancer therapy.

## Introduction

Worldwide, esophageal cancer was the seventh most common cancer and the sixth leading cause of cancer-related deaths, with an estimated 572,000 new cases (3% of all cancers) and 509,000 deaths (5% of all cancers) in 2018 [1]. The prognosis of esophageal cancer is poor, with an 18% overall 5-year survival [2]. There are two main histological subtypes: esophageal squamous cell carcinoma (ESCC) and adenocarcinoma. In the Western countries, adenocarcinoma is predominant, but in Asia and Sub-Saharan Africa, including Japan, ESCC accounts for approximately 90% of esophageal cancer cases [1–3]. ESCC is characterized by a high incidence of lymph node metastasis (37%), which closely correlates poor prognosis [4]. Even among ESCCs localized in the submucosa, 34% of the cases have lymph node metastases, and 22% of the cases have recurrences [5]. A better understanding of the molecular pathogenesis of ESCC is thus necessary to establish novel therapies and biomarkers for this cancer.

Recent evidence indicates that not only the molecular mechanisms that function in tumor cells themselves but also those that function in their surrounding environment hold the key to tumor development in many types of cancer, including ESCC [6, 7]. The tumor microenvironment is composed of tumor cells, stromal non-tumor cells (including fibroblasts, monocytes/macrophages, neutrophils, lymphocytes, and vascular cells), and extracellular matrix [6]. Macrophages are one of the most abundant components of the tumor microenvironment, and they are divided into two phenotypes from an oncologic viewpoint: tumor-suppressive (M1) and tumor-supportive (M2) cells. M1 macrophages are classically activated by interferon-gamma and are characterized by high expressions of pro-inflammatory cytokines such as interleukin (IL)-1, IL-6, IL-12, IL-23, and tumor necrosis factor-alpha. M2 macrophages are alternatively activated and are characterized by anti-inflammatory cytokines such as IL-4, IL-10, IL-13, and tissue growth factor-beta [8–11].

In the microenvironment in various malignancies, tumor-associated macrophages (TAMs) are polarized into the M2 phenotype with IL-4/IL-10^high^, IL-12^low^, cell surface receptors including scavenger receptors CD163 and CD204, and a mannose receptor CD206 [8, 10, 11]. In various malignancies, higher numbers of infiltrating TAMs were reported to be associated with worse prognosis by promoting tumor cell proliferation, invasion, angiogenesis, metastasis formation, and immune suppression [9, 11].

We have demonstrated that a high number of infiltrating CD204^+^ TAMs is associated with higher histological grade, deeper invasion, lymph node metastasis, higher pathological stage, lymphatic invasion, vascular invasion, microvascular density, and poor disease-free survival in ESCC [12]. To explore the functions of TAMs in ESCC, we had compared gene expression profiles between peripheral blood monocyte (PBMo)-derived macrophages and TAM-like macrophages stimulated with the conditioned media of ESCC cell lines by performing a cDNA microarray analysis [13]. Among the upregulated molecules in the TAM-like macrophages, we observed that growth differentiation factor 15, neural cell adhesion molecule, and CXC chemokine ligand 8 (CXCL8, also known as IL-8) contribute to the tumor cell migration, progression, and poor prognosis of ESCC [13–15].

In the present study, we focused on one of the genes that was upregulated in TAM-like macrophages: *CC chemokine ligand 3* (*CCL3*). CCL3, also known as macrophage inflammatory protein-1α (MIP-1α), is a member of the CC chemokine family. CC chemokine receptor 1 (CCR1) and CC chemokine receptor 5 (CCR5) are receptors of CCL3 [16]. The involvement of CCL3 was reported in the progression of various malignancies. For example, the CCL3–CCR1 axis has been associated with the progression of hepatocellular carcinoma [17], whereas the CCL3–CCR5 axis has been associated with osteolysis in multiple myeloma [18, 19], lung metastasis in murine renal cell carcinoma [20], cell migration, and invasion in chondrosarcoma [21], infiltration of cancer-associated fibroblasts in murine colitis-associated colorectal carcinoma [22], and angiogenesis in osteosarcoma [23]. Both the CCL3–CCR1 and CCL3–CCR5 axes are associated with leukemogenesis in chronic myeloid leukemia [24, 25] and the progression of oral squamous cell carcinoma [26, 27].

CCR5 is a chemokine receptor and a member of the G-protein coupled receptor family that is usually expressed in memory T lymphocytes, macrophages, dendritic cells, platelets, neurons, astrocytes, fibroblasts, smooth muscle cells, and capillary endothelial cells [16, 28–30]. CCR5 is not only a receptor for CCL3 but also a receptor for the following members of the CC chemokine family: CCL4, CCL5, CCL8, and CCL3 like-1 (CCL3L1) [29, 30]. CCR5 is expressed in various neoplastic cells including oral squamous cell carcinoma [27], murine renal cell carcinoma [20], chondrosarcoma [21], osteosarcoma [23], breast cancer [31–34], pancreatic cancer [35], salivary adenoid cystic carcinoma [36], and Hodgkin lymphoma [37]. CCR5 expressed in neoplastic cells contributes to cell migration, invasion and tumor progression not only via the CCL3–CCR5 axis [20, 21, 23, 27], but also via the CCL5–CCR5 axis in breast cancer, pancreatic cancer, salivary adenoid cystic carcinoma, Hodgkin lymphoma, and ESCC [31–38]. CCL5–CCR5 and/or CCL8–CCR5 axes are also involved in accumulation of CCR5^+^ regulatory T cells in tumor microenvironment in melanoma, breast cancer, and ovarian cancer [39–41]. However, the roles of CCL3–CCR5 axis in ESCC have not been revealed; therefore, we conducted the present study to address these profiles.

## Materials and methods

### ESCC cell lines and cell cultures

We purchased three ESCC cell lines from the RIKEN BioResource Center (Tsukuba, Japan): TE-8 (moderately differentiated ESCC), TE-9 (poorly differentiated ESCC), and TE-15 (well differentiated ESCC). Het-1A, a normal human esophageal squamous epithelial cell line immortalized SV40-T antigen transfection, was purchased from the American Type Culture Collection® (Manassas, VA) [42]. The individuality of the TE series ESCC cell lines was confirmed by a short tandem repeat analysis at RIKEN and at the Cell Resource Center for Biomedical Research, Institute of Development, Aging and Cancer, Tohoku University (Sendai, Japan) [43]. All three ESCC cell lines were confirmed to be mycoplasma-negative by a Venor® Gem Classic Mycoplasma Detection kit (Minerva Biolabs, Berlin, Germany) and cultured in RPMI-1640 medium (FUJIFILM Wako Pure Chemical, Osaka, Japan) supplemented with 10% fetal bovine serum (FBS) (Sigma-Aldrich, St. Louis, MO) and 1% antibiotic-antimycotic (Invitrogen, Carlsbad, CA). The Het-1A cell line was confirmed to be free of human pathogenic virus using a PCR-based assay for human immunodeficiency virus (HIV), hepatitis B virus, human papilloma virus, Epstein-Barr virus, and cytomegalovirus and cultured in BEGM™ Bronchial Epithelial Cell Growth Medium BulletKit™ (#CC-3170; Lonza, Walkersville, MD).

The conditioned medium of the TE series ESCC cell lines (TECM; i.e., TE-8CM, TE-9CM, and TE-15CM) were prepared by plating 5 × 10^6^ tumor cells in 10 ml of complete medium on 100-mm dishes for 24 h. The medium was then changed to complete Dulbecco’s modified Eagle’s medium (DMEM; FUJIFILM Wako Pure Chemical) supplemented with 10% human AB serum (Lonza). Two days later, the supernatants were harvested, centrifuged, and stored in aliquots at −80 °C.

### Macrophage and TAM-like macrophage cultures

Peripheral blood mononuclear cells (PBMCs) were obtained from healthy volunteer donors with written informed consents. CD14^+^ PBMos were purified from PBMCs by positive selection using an auto MACS® Pro Separator (Miltenyi Biotec, Bergisch Gladbach, Germany). Then, 5 × 10^6^ PBMos were cultured on six-well plates with macrophage-colony stimulating factor (25 ng/ml; R&D Systems, Minneapolis, MN) for 6 days for the induction of macrophages differentiation. PBMo-derived macrophages were treated with 50% TECM for 2 days for the induction of TAM-like polarization. We named the TAM-like macrophages polarized by TE-8CM, TE-9CM, and TE-15CM as, respectively, TAM8, TAM9, and TAM15 cells.

### Reagents

Recombinant human CCL3/MIP-1α protein (rhCCL3, catalog #270-LD), the neutralizing antibody against CCL3 (#AF-270), and normal goat IgG control (#AB-108-C) were purchased from R&D Systems. The inhibitors against PI3K (LY294002, #9901) and MEK (PD98059, #9900) were purchased from Cell Signaling Technology (Danvers, MA). The CCR5 antagonist, Maraviroc (#PZ0002) was purchased from Sigma-Aldrich.

### *CCR5* knockdown by small interfering RNA (siRNA)

For the CCR5 knockdown by siRNA, 5 × 10^5^ TE-8, TE-9, and TE-15 cells on 60 mm dishes were transfected by 20 nM siRNA against CCR5 (siCCR5, #sc-35062; Santa Cruz Biotechnology) using Lipofectamine® RNAiMAX (Invitrogen) for 2 days. Control siRNA (Sigma-Aldrich) was used as the negative control (siNC).

### Reverse transcription PCR (RT-PCR) and quantitative RT-PCR

Total RNA was extracted from cultured cells with the use of an RNeasy Mini Kit (Qiagen, Hilden, Germany). Reverse transcription-polymerase chain reaction (RT-PCR) amplifications of *CCL3*, *CCR1*, *CCR5*, *MMP2*, *MMP9*, *VEGFA*, and the internal control gene *GAPDH* were performed. PCR products were subjected to electrophoresis in a 2% agarose gel. The primers used for RT-PCR were: *CCL3*, 5′-TCT GCA TCA CTT GCT GCT GAC AC-3′ (forward), 5′-CAC TCA GCT CCA GGT CGCTGA C-3′ (reverse); *CCR1*, 5′-CAC GGA CAA AGT CCC TTG G-3′ (forward), 5′-CAA AGG CCC TCT CGT TCA C-3′ (reverse); *CCR5*, 5′-GAC ATC CGT TCC CCT ACA AG-3′ (forward), 5′-AGA TGA ACA CCA GTG AGT AGA G-3′ (reverse); *GAPDH*, 5′-ACC ACA GTC CAT GCC ATC AC-3′ (forward), 5′-TCC ACC ACC CTG TTG CTG TA-3′ (reverse).

A quantitative RT-PCR was performed using the following probes: *CCL3* (Hs00234142_m1), *MMP2* (Hs01548727_m1), *MMP9* (Hs00234579_m1), *VEGFA* (Hs00900054_m1), and *GAPDH* (Hs02786624_g1) (Applied Biosystems, Foster City, CA) on an ABI StepOne Real-time PCR system (Applied Biosystems) using TaqMan Gene Expression Master Mix (Applied Biosystems). The threshold cycle (Ct) values were determined by plotting the observed fluorescence against the cycle number. Ct values were analyzed using the comparative threshold cycle method and normalized to those of *GAPDH*. We used the following formula to estimate the relative gene expression levels: relative expression = 2^−(Ct [*target gene*] − Ct [*GAPDH*])^.

### Enzyme-linked immunosorbent assay (ELISA)

For an ELISA of CCL3, 5 × 10^5^ PBMo-derived macrophages, TAM8 cells, TAM9 cells, TAM15 cells, and 2 × 10^6^ of each of the ESCC cell lines were incubated on six-well plates with 2 ml of serum-free DMEM. Two days later, the media were collected and applied to a Quantikine® ELISA Human CCL3 immunoassay (#DMA00; R&D Systems). We determined the optical density (OD) using a Microplate Reader Infinite® 200 PRO (Tecan, Männedorf, Switzerland).

### Western blotting

Cells were lysed in cell lysis buffer (50 mM Tris-HCl pH 7.5, 125 mM NaCl, 5 mM EDTA and 0.1% Triton X-100) containing 1% protease inhibitor and 1% phosphatase inhibitor cocktail (Sigma-Aldrich). The resulting lysates were separated on 5–20% or 15–20% s sodium dodecyl sulfate (SDS) polyacrylamide gels for SDS-polyacrylamide gel electrophoresis (PAGE) and then transferred to a membrane with an iBlot® Gel Transfer Stack (Invitrogen). The membrane was blocked with 5% skim milk and then incubated with the primary antibody at 4 °C overnight and then with secondary antibodies for 90 min at the room temperature. The protein bands were detected with ImmunoStar® Reagents (FUJIFILM Wako Pure Chemical). Densitometric analyses of bands were performed with ImageJ software ver. 1.8.0 (National Institutes of Health, Bethesda, MD).

The primary antibodies were as follows. Rabbit polyclonal antibody against CCR1 (1:250, #ab1681; Abcam, Cambridge, UK), mouse monoclonal antibody against CCR5 (1:100, #sc-32304; Santa Cruz Biotechnology), rabbit polyclonal antibody against CCL3 (1:100, #LS-C384561; LifeSpan BioSciences, Seattle, WA), and the following rabbit monoclonal antibodies (all from Cell Signaling Technology): phosphorylated Akt (Ser473; 1:250, #4060), phosphorylated Akt (Thr308; 1:250, #2965), total Akt (1:500, #9272), phosphorylated ERK1/2 (Thr202/Tyr204; 1:250, #9101), total ERK1/2 (1:500, #9102), and β-actin (1:1000, #4970).

The secondary antibodies were horseradish peroxidase (HRP)-linked donkey anti-rabbit IgG (1:1000, #NA934V) and HRP-linked sheep anti-mouse IgG (1:1000, #NA931V), both were purchased from GE Healthcare Life Sciences (Little Chalfont, UK).

### Immunofluorescence (IF)

For the IF examination, 1 × 10^5^ cultured cells on coverslips were fixed with methanol for 10 min at −20 °C and incubated with primary antibodies against CCR5 (1:25, #sc-32304; Santa Cruz Biotechnology), CCL3 (1:100, #LS-C384561; LifeSpan BioSciences), and CD204 (1:100, #SRA-E5; TransGenic, Kobe, Japan) at 4 °C overnight. The cells were then incubated with AlexaFluor-488® conjugated donkey anti-rabbit secondary antibody, AlexaFluor-488® conjugated donkey anti-mouse secondary antibody, and Cy3-conjugated donkey anti-mouse IgG secondary antibody (1:200; Jackson ImmunoResearch Laboratories, West Grove, PA). The nuclei were stained by 4′,6-diamidino-2-phenylindole (DAPI, 1:1000; Vector Laboratories, Burlingame, CA). Images were taken with a Zeiss LSM 700 laser-scanning microscope and analyzed using the LSM software ZEN 2009 (Carl Zeiss, Oberkochen, Germany).

### Transwell migration and invasion assays

For the migration assay, 2 × 10^5^ TE-8 cells or 1 × 10^5^ TE-9 cells in 300 µl of medium with 0.1% FBS were placed in the upper transwell inserts with an 8-µm pore filter (BD Falcon, Lincoln Park, NY) in 24-well plates. For the invasion assay, the same number of TE-8 or TE-9 cells in the same medium were placed in the inserts of a Corning® BioCoat™ Matrigel® Invasion Chamber (Corning, Tewksbury, MA) in 24-well plates, and then 800 µl of medium containing 100 ng/ml rhCCL3 or 1 × 10^5^ TAMs was placed in the lower chamber. Next, 20 µM LY294002, 20 µM PD98059, or 20 µg/ml Maraviroc was added to the upper chambers, and the neutralizing antibody against CCL3 (400 ng/ml) was added to the lower chambers. The plates were then incubated for 24 h (for the migration assay) or 48 h (for the invasion assay) at 37 °C in 5% CO_2_. The cells were fixed in methanol for 1 min and stained with Diff-Quik® (Sysmex, Kobe, Japan). Cells on the upper side of the filters were removed with cotton-tipped swabs. Five images at ×200 magnification were obtained from each membrane with a CCD camera (Olympus, Tokyo, Japan), and the number of cells was counted. The percent migration or invasion was calculated by dividing the number of cells by that in the negative control (without rhCCL3, co-cultured TAMs, inhibitors, Maraviroc, or the neutralizing antibody of CCL3).

### Cell survival and growth assays

For the cell survival assay, 1 × 10^4^ of each of the ESCC cell lines were plated in 96-well plates with 100 µl of serum-free medium containing 10 or 100 ng/ml rhCCL3. For the cell growth assay, 5 × 10^3^ of each of the ESCC cell lines were plated in medium supplemented with 0.1% FBS. The plates were incubated for 72 h, and an MTS assay was performed: 20 µl CellTiter 96® AQueous One Solution Reagent (Promega, Madison, WI) was added to the medium, and then the OD at 492 nm was determined.

### ESCC tissue samples and immunohistochemistry

We examined a total of 68 human ESCC samples that were surgically resected at Kobe University Hospital (Kobe, Japan) during the years from 2005 to 2010. None of the ESCC patients had received neoadjuvant chemotherapy or radiotherapy before surgery. Informed consent for the use of their tissue samples was obtained from all patients, and the study was approved by the Kobe University Institutional Review Board. All resected specimens were fixed with 10% formalin, embedded in paraffin wax, and sliced at 4-µm thickness. Each sample was categorized based on the 11th edition of Japanese Classification of Esophageal Cancer and the 8th edition of Union for International Cancer Control (UICC) TNM Classification of Malignant Tumours [44–46].

Immunohistochemistry was performed using EnVision™+ Dual Link System-HRP with 3,3′-diaminobenzidine (Dako Cytomation, Glostrup, Denmark). The primary antibodies were CCL3 (1:200, #LS-C384561; LifeSpan BioSciences), CCR5 (1:25, #sc-32304; Santa Cruz Biotechnology), CD68 (1:100, #Kp-1; Dako), CD163 (1:100, #10D6; Novocastra, Newcastle upon Tyne, UK), CD204 (1:50, #SRA-E5; TransGenic), and CD34 (1:50, #NU-4A1, Nichirei, Tokyo, Japan).

CCL3 expression was evaluated based on the staining intensity in the cancer nests as “low” (i.e., with intensity that is weaker than or equal to that of noncancerous epithelium) and “high” (with stronger intensity than that of noncancerous epithelium). We evaluated the CCR5 expression based on the staining intensity in the cancer nests as “low” (intensity weaker than or equal to that in the muscularis propria) and “high” (stronger than the intensity in muscularis propria). The infiltrating number of TAMs was evaluated by CD68, CD163, and CD204 immunohistochemistry as previously described [12]. Microvascular density was evaluated by CD34 immunohistochemistry as previously described [12]. The evaluation and scoring were performed by three independent pathologists (authors TK, YK, and HY).

### Statistical analyses

All experiments were performed in triplicate and conducted independently at least three times. The results are expressed as the mean ± standard error (SEM), and statistical significance was analyzed by two-sided Student’s *t* test. The relationships between clinicopathological factors and immunohistochemistry were estimated by *χ*^2^-test. Overall, the disease-free and cause-specific survival curves were estimated by the Kaplan–Meier method and compared by log-rank test. The significance of parameters in the univariate and multivariate analyses was tested using the Cox proportional hazard regression model. A *p* value < 0.05 was considered significant. The statistical analyses were carried out using SPSS Statistics ver. 22 (IBM, Chicago, IL).

## Results

### The TAM-like macrophages expressed CCL3

**Fig. 1:**
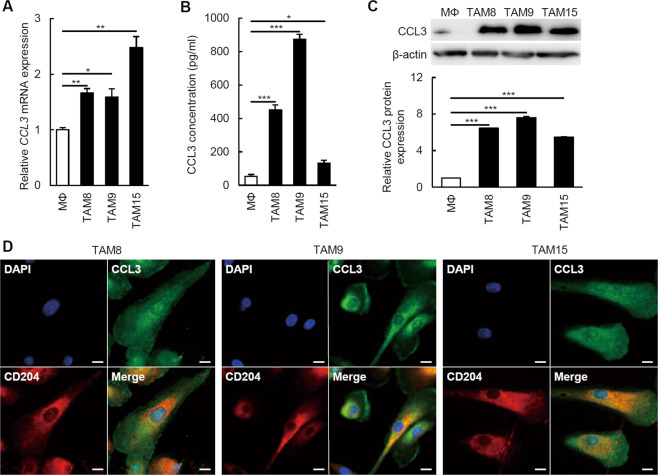
CCL3 expression in PBMo-derived macrophages and TAM-like macrophages. **a** The *CCL3* mRNA expression levels of macrophages (MΦ) and TAM-like macrophages (TAM8, TAM9, and TAM15) were determined by quantitative RT-PCR and normalized to *GAPDH* expression. Data are mean ± SEM (*n* = 3, *p < 0.05, **p < 0.01). Compared with MΦ, TAM8, TAM9, and TAM15 expressed significantly higher levels of *CCL3* mRNA. **b** The CCL3 concentration in conditioned medium of MΦ, TAM8, TAM9, and TAM15. Protein levels were measured by ELISA. Results are mean ± SEM (*n* = 4, *p < 0.05, ***p < 0.001). TAM8, TAM9, and TAM15 secreted significantly more CCL3 compared with MΦ. **c** Western blotting was conducted with total protein from MΦ, TAM8, TAM9, and TAM15 using antibodies against CCL3 and β-actin. Higher levels of CCL3 expression was detected in TAM8, TAM9, and TAM15 compared with MΦ. The densitometric analyses were performed by ImageJ software. Results are mean ± SEM (***p < 0.001). **d** The co-expression of CCL3 and CD204 in TAM8, TAM9, and TAM15. Double immunofluorescence was performed using antibodies against CCL3 (*green*) and CD204 (*red*). TAM8, TAM9, and TAM15 expressed both CCL3 and CD204. Nuclei were stained by DAPI (*blue*). Magnification: ×600. Scale bar: 10 µm.

We first confirmed the expression level of CCL3 in TAM-like macrophages. Compared with the *CCL3* mRNA expression level in the PBMo-derived TAM-like macrophages (1.0 ± 0.0-folds), the expression levels were significantly higher in the PBMo-derived TAM-like macrophages polarized by TE-8CM (TAM8 cells, 1.7 ± 0.1-fold, *p* = 0.002), TE-9CM (TAM9 cells, 1.6 ± 0.1-fold, *p* = 0.018), and TE-15CM (TAM15 cells, 2.5 ± 0.2-fold, *p* = 0.002) (Fig. 1a). The ELISA results showed that the CCL3 concentrations of the conditioned medium of TAM8 cells (451.0 ± 29.1 pg/ml, *p* < 0.001), TAM9 cells (873.0 ± 31.2 pg/ml, *p* < 0.001), and TAM15 cells (131.5 ± 17.3 pg/ml, *p* = 0.016) were significantly higher than that of the PBMo-derived macrophages (52.7 ± 11.3 pg/ml) (Fig. 1b). The western blotting using antibodies against CCL3 detected much higher CCL3 expressions in the TAM8 cells (6.4 ± 0.0-fold, *p* < 0.001), TAM9 cells (7.6 ± 0.1-fold, *p* < 0.001), and TAM15 cells (5.5 ± 0.1-fold, *p* < 0.001) compared with the PBMo-derived macrophages (1.0 ± 0.0-fold) (Fig. 1c). IF detected expression of CCL3 mainly in the cytoplasm of CD204^+^ TAM8, TAM9, and TAM15 cells (Fig. 1d).

### The ESCC cell lines expressed CCR5 and CCL3

**Fig. 2:**
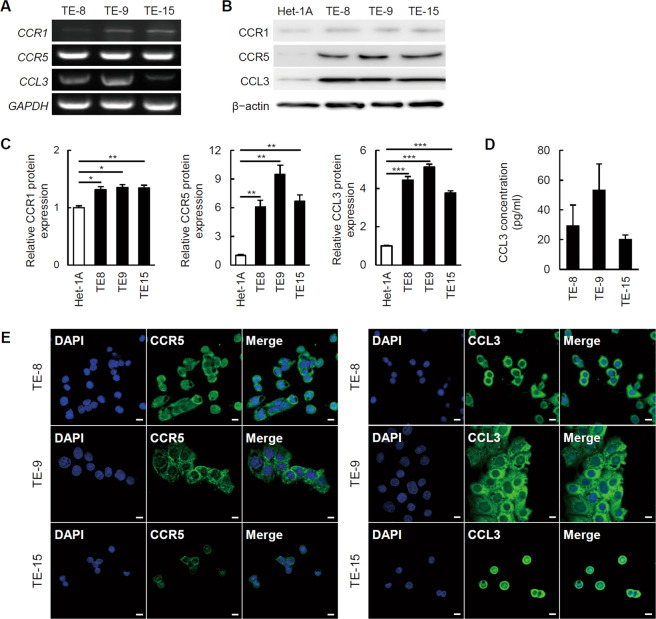
The expression of CCR1, CCR5, and CCL3 in the ESCC cell lines. **a** *CCR1*, *CCR5*, and *CCL3* mRNA expressions in TE-8, TE-9, and TE-15 cells were detected by RT-PCR. The results of western blotting (**b**) and their densitometric analyses (**c**, mean ± SEM, *p < 0.05, **p < 0.01, ***p < 0.01). CCR1, CCR5, and CCL3 protein expressions in TE-8, TE-9, and TE-15 cells were significantly higher than those of Het-1A cells. However, CCR1 expression levels in TE-8, TE-9, and TE-15 cells were relatively lower than CCL3 and CCR5 expression levels. **d** CCL3 concentration in conditioned medium of TE-8, TE-9, and TE-15 cells. Protein levels were measured by ELISA. Results are mean ± SEM (*n* = 4). CCL3 secretion was detected from TE-8, TE-9, and TE-15 cells. **e** Immunofluorescence using anti-CCR5 or anti-CCL3 (*green*) antibody on TE-8, TE-9, and TE-15 cells. Nuclei were stained by DAPI (*blue*). CCL3 and CCR5 were expressed in TE-8, TE-9, and TE-15 cells. Magnification: ×400. Scale bar: 10 µm.

We next explored the expression levels of CCR1 and CCR5, which are CCL3 receptors. CCR1 and CCR5 expressions in the TE-8, TE-9, and TE-15 cells were confirmed by RT-PCR (Fig. 2a). Moreover, the western blotting results showed that the expression levels of CCR5 in the TE-8 (6.1 ± 0.7-fold, *p* = 0.004), TE-9 (9.5 ± 1.0-fold, *p* = 0.002), and TE-15 cells (6.7 ± 0.6-fold, *p* = 0.002) were significantly higher than that in the Het-1A cells (1.0 ± 0.1-fold). The expression levels of CCR1 in TE-8 (1.3 ± 0.1-fold, *p* = 0.017), TE-9 (1.4 ± 0.1-fold, *p* = 0.010), and TE-15 cells (1.3 ± 0.0-fold, *p* = 0.008) were also significantly higher than that in the Het-1A cells (1.0 ± 0.0-fold), but relatively much lower than the CCR5 expression levels in the three ESCC cell lines (Fig. 2b, c).

The RT-PCR results demonstrated that not only TAMs but also the three ESCC cell lines expressed CCL3 (Fig. 2a). The western blotting results showed that the expression levels of CCL3 in the TE-8 (4.4 ± 0.2-fold, *p* < 0.001), TE-9 (5.1 ± 0.2-fold, *p* < 0.001), and TE-15 cells (3.8 ± 0.1-fold, *p* < 0.001) were also significantly higher than that in the Het-1A cells (1.0 ± 0.0-fold) (Fig. 2b, c). The ELISA results also confirmed the secretion of CCL3 from the ESCC cell lines (Fig. 2d).

We then performed an IF study using anti-CCR5 or anti-CCL3 antibodies on TE-8, TE-9, and TE-15 cells (Fig. 2e). CCR5 expression was detected in the cellular membrane and cytoplasm of TE-8, TE-9, and TE-15 cells, and CCL3 expression was detected in the cytoplasm of TE-8, TE-9, and TE-15 cells.

### CCL3 activated Akt and ERK signaling pathways via CCR5 in ESCC cell lines

**Fig. 3:**
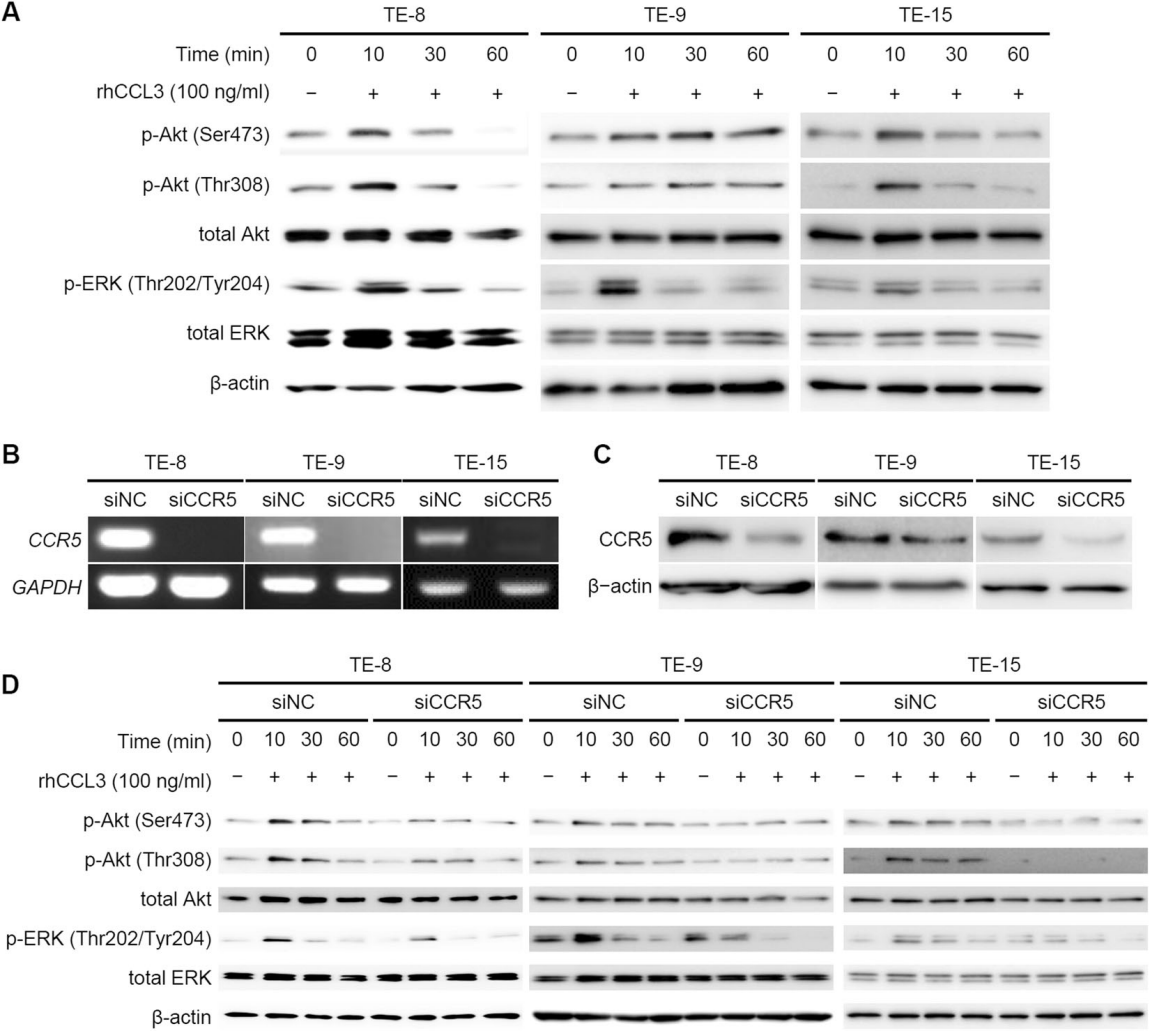
Signaling pathways in ESCC cell lines treated with rhCCL3. **a** First, 5 × 10^5^ TE-8, TE-9, and TE-15 cells under serum-free condition were treated with 100 ng/ml rhCCL3 for 10, 30, and 60 min. Western blotting was conducted with total protein extracted from the ESCC cell lines using antibodies against total Akt, phosphorylated (p-)Akt (Ser473), p-Akt (Thr308), total ERK, p-ERK (Thr202/Tyr204), and β-actin. Akt and ERK were phosphorylated at 10 min after rhCCL3 treatment in TE-8, TE-9, and TE-15 cells. The results of densitometric analyses were shown in Fig S1. **b**–**d** First, 5 × 10^5^ TE-8, TE-9, and TE-15 cells was transfected by 20 nM siRNA against CCR5 (siCCR5) and negative control siRNA (siNC) for 2 days. The CCR5 knockdown of the ESCC cell lines was confirmed by RT-PCR (**b**) and western blotting using anti-CCR5 (**c**). Western blotting was conducted with total protein extracted from the ESCC cell lines transfected siCCR5 and siNC (**d**). The phosphorylations of Akt and ERK stimulated by rhCCL3 was suppressed by transfection with siCCR5 in TE-8, TE-9, and TE-15 cells. The results of densitometric analyses were shown in Fig S2.

We next investigated the effect of CCL3 on the ESCC cell lines. For an exploration of the signaling pathways, we treated TE-8, TE-9, and TE-15 cells with 100 ng/ml of rhCCL3 for 10, 30, and 60 min. We observed the phosphorylations of Akt and ERK at 10 min after the rhCCL3 treatment in TE-8, TE-9, and TE-15 cells (Figs. 3a, S1).

We then silenced CCR5 in TE-8, TE-9, and TE-15 cells with the use of siRNA. CCR5 knockdown was confirmed by RT-PCR and western blotting (Figs. 3b, c; S2A). The western blotting revealed that the phosphorylations of Akt and ERK at 10 min after rhCCL3 treatment were suppressed by the CCR5 knockdown in TE-8, TE-9, and TE-15 (Figs. 3d, S2B). These results suggest a CCL3–CCR5 axis activated Akt and ERK signaling pathways in these ESCC cell lines.

### CCL3–CCR5 axis-induced migration and invasion of ESCC cell lines via Akt and ERK signaling pathways

**Fig. 4:**
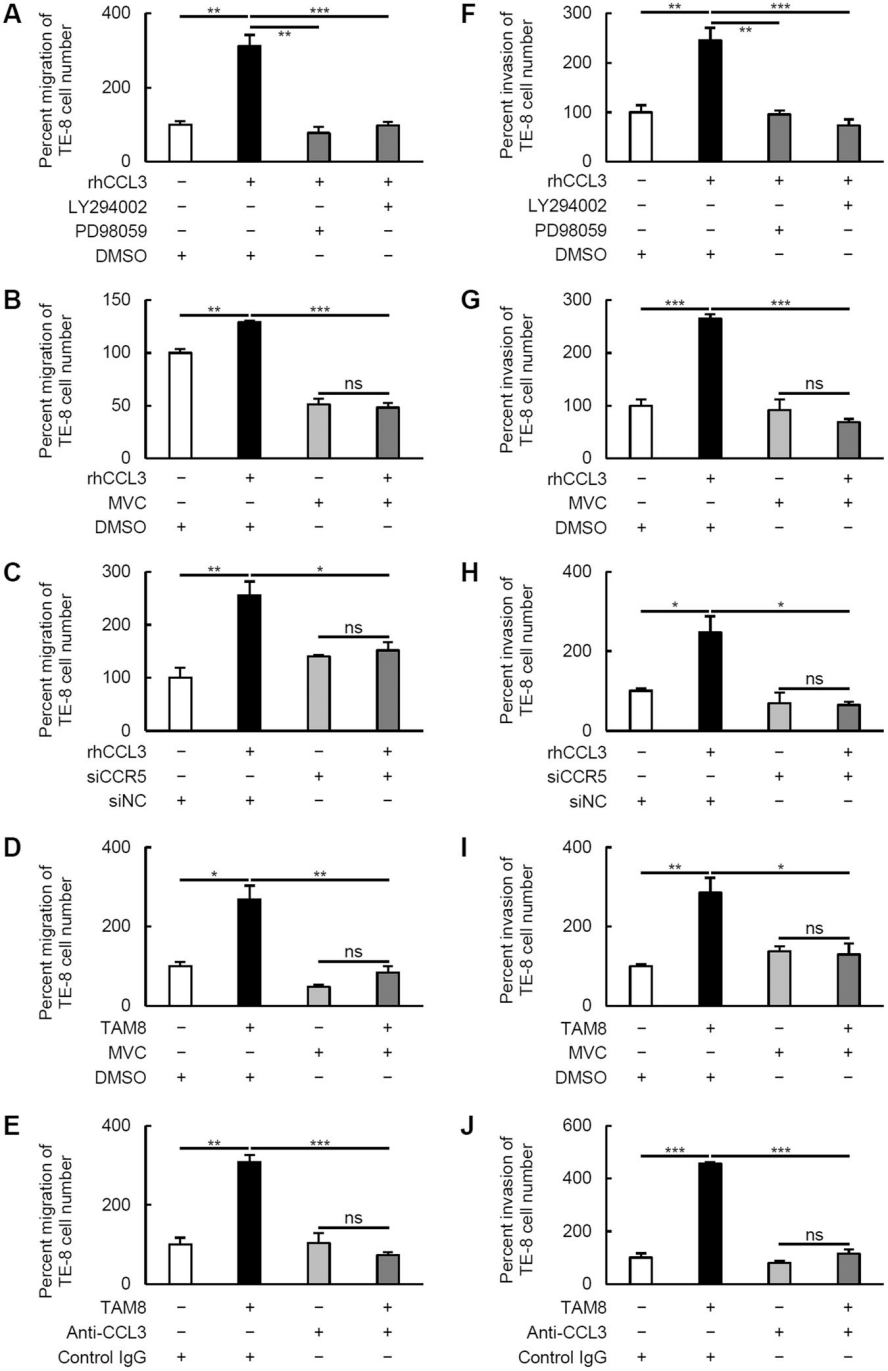
Transwell migration and invasion assays of TE-8 cells. **a**–**e** For the migration assay, 2 × 10^5^ TE-8 cells were plated on the transwell in RPMI-1640 medium containing 0.1% FBS. Then 100 ng/ml rhCCL3 (**a**–**c**) or 1 × 10^5^ TAM8 (**d**, **e**) was added in the lower chamber. The cell inserts were set on 24-well plates for 24 h. For the invasion assay, TE-8 cells were plated on the transwell in the same condition, and rhCCL3 (**f**–**h**) or TAM8 (**I, J**) was added in the lower chamber. The cell inserts were incubated for 48 h. The migrated and invaded cells on the underside of the membrane were stained and counted. Results are mean ± SEM (*n* = 3; *p < 0.05; **p < 0.01; ***p < 0.001; ns not significant). In each assay, the percent migration or invasion was calculated by dividing the number of TE-8 cells by that in the negative control. **a**, **f** 20 µM LY294002 and PD98059 were added on the upper chamber, and 0.2 µl/ml DMSO was added as a negative control. **b**, **d**, **g**, **i** 20 µg/ml Maraviroc (MVC) was added on the upper chamber, and 0.2 µl/ml DMSO was added as a negative control. **c**, **h** 2 × 10^5^ TE-8 cells transfected with 20 nM siCCR5 or siNC were plated. **e**, **j** 400 ng/ml CCL3 neutralizing antibody (anti-CCL3) was added on the lower chamber, and 400 ng/ml control IgG was added as a negative control.

To explore the roles of the CCL3–CCR5 axis in the ESCC cell lines, we focused on cell migration and invasion. Our findings first revealed that rhCCL3 significantly promoted the migration and invasion of TE-8 cells, and the migration and invasion were significantly suppressed by the inhibition of PI3K/Akt and MEK/ERK pathways using LY294002 and PD98059, respectively (Fig. 4a, f; representative images are shown in Fig. S4A, B). Maraviroc and CCR5 knockdown were also effective for suppressing the rhCCL3-mediated migration and invasion (Fig. 4b, c, g, h). We then performed the same assays using a co-culture with TAM8 instead of rhCCL3 (Fig. 4d, e, i, j), and observed that TAM8 also significantly promoted the migration and invasion of TE-8 cells, and that the migration and invasion were significantly suppressed by Maraviroc and neutralizing antibody against CCL3.　When these assays were performed using TE-9 and TAM9 cells instead of TE-8 and TAM8 cells, almost the same results was observed (Fig. S3; representative images are shown in Fig. S4C, D).

Next, we investigated whether the CCL3 derived from ESCC cells could promote migration and invasion via CCR5. TE-8 and TE-9 cells were mono-cultured in the upper chamber, and Maraviroc or the neutralizing antibody against CCL3 was added to the lower chamber. The migration of TE-8 cells (Fig. S5A; 100.0 ± 8.6 vs. 81.6 ± 7.7%, *p* = 0.213), and the invasion of TE-8 (Fig. S5E; 100.0 ± 34.3 vs. 83.5 ± 13.3%, *p* = 0.684) and TE-9 cells (Fig. S5F; 100.0 ± 11.1 vs. 87.8 ± 5.0%, *p* = 0.383) were slightly suppressed by the neutralizing antibody without statistical significance. The migration of TE-9 cells was not suppressed by the neutralizing antibody (Fig. S5B; 100.0 ± 4.3 vs. 101.1 ± 7.2%, *p* = 0.914). The migration of TE-9 cells (Fig. S5D; 100.0 ± 13.8 vs. 72.7 ± 6.2%, *p* = 0.154), and the invasion of TE-8 (Fig. S5G; 100.0 ± 17.5 vs. 73.9 ± 9.0%, *p* = 0.289) and TE-9 cells (Fig. S5H; 100.0 ± 15.7 vs. 75.2 ± 9.9%, *p* = 0.272) were slightly suppressed by Maraviroc without statistical significance. The migration of TE-8 cells was not suppressed by Maraviroc (Fig. S5C; 100.0 ± 3.4 vs. 106.5 ± 4.7%, *p* = 0.367). Treatment with rhCCL3 did not show any significant effect on the survival or growth of TE-8, TE-9, and TE-15 cells (Fig. S6). These results suggest that a CCL3–CCR5 axis promotes the migration and invasion of ESCC independently of cell survival and growth.

### The CCL3–CCR5 axis upregulated the MMP-2 and VEGF-A expressions in the ESCC cell lines

**Fig. 5:**
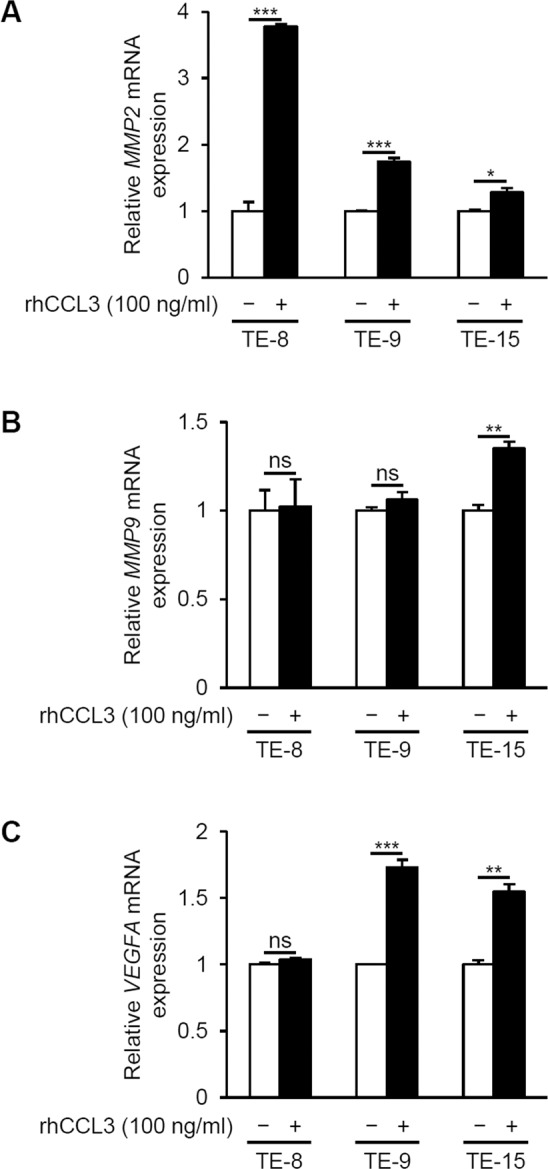
The expressions of matrix metalloproteases and an angiogenic factor VEGF-A in the ESCC cell lines treated with rhCCL3. First, 5 × 10^5^ TE-8, TE-9, or TE-15 cells under serum-free conditions were treated with 100 ng/ml rhCCL3 for 48 h. **a** *MMP2*, **b** *MMP9*, and **c** *VEGFA* mRNA expression levels of the ESCC cell lines were determined by quantitative RT-PCR and normalized to *GAPDH* expression. Data are mean ± SEM (*n* = 3, *p < 0.05; **p < 0.01; ***p < 0.001; ns not significant).

After observing that a CCL3–CCR5 axis promoted the migration and invasion of the three ESCC cell lines via PI3K/Akt and MEK/ERK pathways as described above, we investigated whether CCL3 upregulated the expressions of matrix metalloprotease-2 (MMP-2) and matrix metalloprotease-9 (MMP-9), which are well known to contribute to cell invasion by extracellular matrix remodeling in the tumor microenvironment [6]. We focused on the expression of vascular endothelial growth factor A (VEGF-A) as a surrogate marker of angiogenesis. For the determination of the *MMP2*, *MMP9*, and *VEGFA* mRNA expression levels, we treated TE-8, TE-9, and TE-15 cells with 100 ng/ml of rhCCL3 for 48 h. *MMP2* mRNA was significantly upregulated in the TE-8 cells (1.0 ± 0.1 vs. 3.8 ± 0.0-fold, *p* < 0.001), TE-9 cells (1.0 ± 0.0 vs. 1.8 ± 0.1-fold, *p* < 0.001), and TE-15 cells (1.0 ± 0.0 vs. 1.3 ± 0.1-fold, *p* = 0.017) at 48 h after rhCCL3 treatment (Fig. 5a). *MMP9* mRNA was significantly upregulated in the TE-15 cells (1.0 ± 0.0 vs. 1.4 ± 0.0-fold, *p* = 0.002), but not in TE-8 cells (1.0 ± 0.1 vs. 1.0 ± 0.2-fold, *p* = 0.911) or TE-9 cells (1.0 ± 0.0 vs. 1.1 ± 0.0-fold, *p* = 0.263) (Fig. 5b). *VEGFA* mRNA was significantly upregulated in TE-9 cells (1.0 ± 0.0 vs. 1.7 ± 0.1-fold, *p* < 0.001) and TE-15 cells (1.0 ± 0.0 vs. 1.5 ± 0.1-fold, *p* = 0.001), but not in TE-8 cells (1.0 ± 0.0 vs. 1.0 ± 0.0-fold, *p* = 0.089) (Fig. 5c). Next, we investigated the *MMP2*, *MMP9*, and *VEGFA* mRNA expression levels after LY294002 or PD98059 treatment. TE-8 and TE-15 cells were treated with 100 ng/ml of rhCCL3 and 20 µM of LY294002 or PD98059 for the first 24 h, then they were treated with only rhCCL3 for the next 24 h. TE-9 cells were treated with 100 ng/ml of rhCCL3 and 20 µM of LY294002 or PD98059 for 48 h. The upregulated mRNA expression of *MMP2* and *VEGFA* was suppressed by both LY294002 and PD98059 treatment (Fig. S7A, C). In contrast, the *MMP9* mRNA expression in TE-15 was upregulated by LY294002 or PD98059 with rhCCL3 treatment (Fig. S7B). Thus, the treatment with rhCCL3 upregulated the expressions of MMP-2 and VEGF-A in multiple ESCC cell lines via PI3K/Akt and MEK/ERK pathways.

### CCL3 and/or CCR5 expression in human ESCC tissues is correlated with poor patient prognosis

**Fig. 6:**
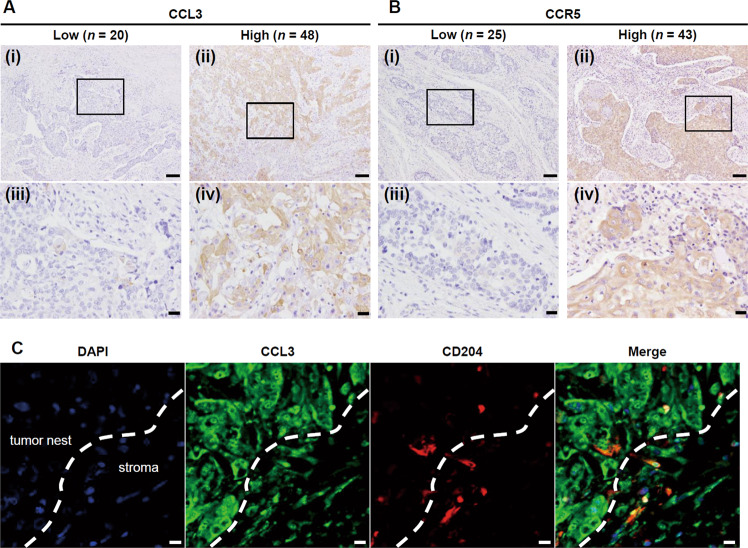
The expression of CCL3 and CCR5 in the human ESCC tissue samples. **a** Representative CCL3 immunoreactivities in human ESCC tissues. The immunoreactivity in the tumor nests was assessed and divided into low (*n* = 20) and high (*n* = 48) groups based on the staining intensity. Typical images are shown: low expression (i, iii) and high expression (ii, iv). Areas delimited by squares in (i) and (ii) (magnification: ×100; scale bar: 100 µm) are showed in higher magnification in (iii) and (iv) (magnification: ×400; scale bar: 20 µm), respectively. **b** Representative CCR5 immunoreactivities in human ESCC tissues. Immunoreactivity in the tumor nest was assessed and divided into low (*n* = 25) and high (*n* = 43) groups based on the staining intensity. Typical images are shown: low expression (i, iii) and high expression (ii, iv). Areas delimited by squares in (i) and (ii) (magnification: ×100; scale bar: 100 µm) are showed in higher magnification in (iii) and (iv) (magnification: ×400; Scale bar: 20 µm), respectively. **c** Double immunofluorescence was performed using antibodies against CCL3 (*green*) and CD204 (*red*). CCL3 was expressed in both cancer cells and CD204^+^ TAMs. Nuclei were stained by DAPI (*blue*). White dotted line indicates the edge of the tumor nest. Magnification: ×400. Scale bar: 10 µm.

Lastly, we investigated the expressions of CCL3 and CCR5 in the human ESCC tissues, and we determined the correlations between these expressions and each of several clinicopathological factors and patient prognosis by immunohistochemistry. Both CCL3 and CCR5 immunoreactivities were detected diffusely in the tumor nests. The CCL3 and CCR5 expression levels in the tumor nests were assessed and divided into low and high groups based on the staining intensity (Fig. 6a, b). Double IF of CCL3 and CD204 confirmed that CCL3 was expressed both by TAMs and cancer cells (Fig. 6c).

A high expression of CCL3 did not significantly correlate with any of the clinicopathological factors, whereas a high expression of CCR5 was significantly correlated with deeper invasion (*p* = 0.016), presence of vascular invasion (*p* = 0.029), higher pathological stage (*p* = 0.041), higher numbers of infiltrating CD204^+^ TAMs (*p* = 0.006), and higher microvascular density (*p* = 0.006). Because we detected a significant correlation between a high expression of CCL3 and CCR5 (*p* = 0.010), we divided the patients into two groups: the patients with high expressions of both CCL3 and CCR5 (H/H group, *n* = 35), and the other patients (non-H/H group, *n* = 33). The H/H group was significantly correlated with higher pathological stage (*p* = 0.044) (Table 1).

**Table 1:** Correlations between CCL3 and/or CCR5 expression and clinicopathological factors.

	*n*	CCL3 expression			CCR5 expression			CCL3 and CCR5 expression		
		Low (*n* = 20)	High (*n* = 48)	*p* value	Low (*n* = 25)	High (*n* = 43)	*p* value	Non-H/H (*n* = 33)	H/H (*n* = 35)	*p* value
Age										
<65	32	10	22	0.754	12	20	0.906	15	17	0.977
≥65	36	10	26		13	23		17	19	
Sex										
Female	14	4	10	0.938	6	8	0.596	9	5	0.147
Male	54	16	38		19	35		23	31	
Histological grade^a^										
IS + WD	15	5	10	0.706	5	10	0.755	7	8	0.973
MD + PD	53	15	38		20	33		25	28	
Depth of invasion^b^										
Tis + T1	48	15	33	0.606	22	26	0.016*	26	22	0.069
T2 + T3	20	5	15		3	17		6	14	
Lymphatic invasion										
Negative	37	12	25	0.550	17	20	0.086	19	18	0.438
Positive	31	8	23		8	23		13	18	
Vascular invasion										
Negative	43	12	31	0.721	20	23	0.029*	22	21	0.374
Positive	25	8	17		5	20		10	15	
Lymph node metastasis										
Negative	43	16	27	0.064	19	24	0.096	24	19	0.058
Positive	25	4	21		6	19		8	17	
Pathological stage^b^										
0 + I	38	14	24	0.130	18	20	0.041*	22	16	0.044*
II + III + IV	30	6	24		7	23		10	20	
CD68^+^ cells^c^										
Low	35	10	25	0.876	13	22	0.947	15	20	0.475
High	33	10	23		12	21		17	16	
CD163^+^ cells^c^										
Low	34	9	25	0.595	15	19	0.209	17	17	0.627
High	34	11	23		10	22		15	19	
CD204^+^ cells^c^										
Low	34	11	23	0.595	18	16	0.006**	19	15	0.145
High	34	9	25		7	27		13	21	
Microvascular density^d^										
Low	34	11	23	0.595	18	16	0.006**	18	16	0.331
High	34	9	25		7	27		14	20	
CCR5 expression										
Low	25	12	13	0.010*						
High	43	8	35							

Data were analyzed by *χ*^2^-test.

*p  <  0.05, **p  <  0.01.

^a^According to the 11th edition of Japanese Classification of Esophageal Cancer: *IS* squamous cell carcinoma (SCC) in situ/high grade intraepithelial neoplasia, *WD* well differentiated SCC, *MD* moderately differentiated SCC, *PD* poorly differentiated SCC [44, 45].

^b^According to the 8th edition of the TNM classification: *Tis* carcinoma in situ/high grade dysplasia, *T1* tumor invades lamina propria, muscularis mucosae or submucosa, *T2* tumor invades muscularis propria, *T3* tumor invades adventitia [46].

^c^The patients were divided into low and high groups based on the median number of infiltrating CD68^+^, CD163^+^, or CD204^+^ macrophage in tumor cell nests and the tumor stroma [12].

^d^The patients were divided into low and high groups based on the median microvascular density [12].

**Fig. 7:**
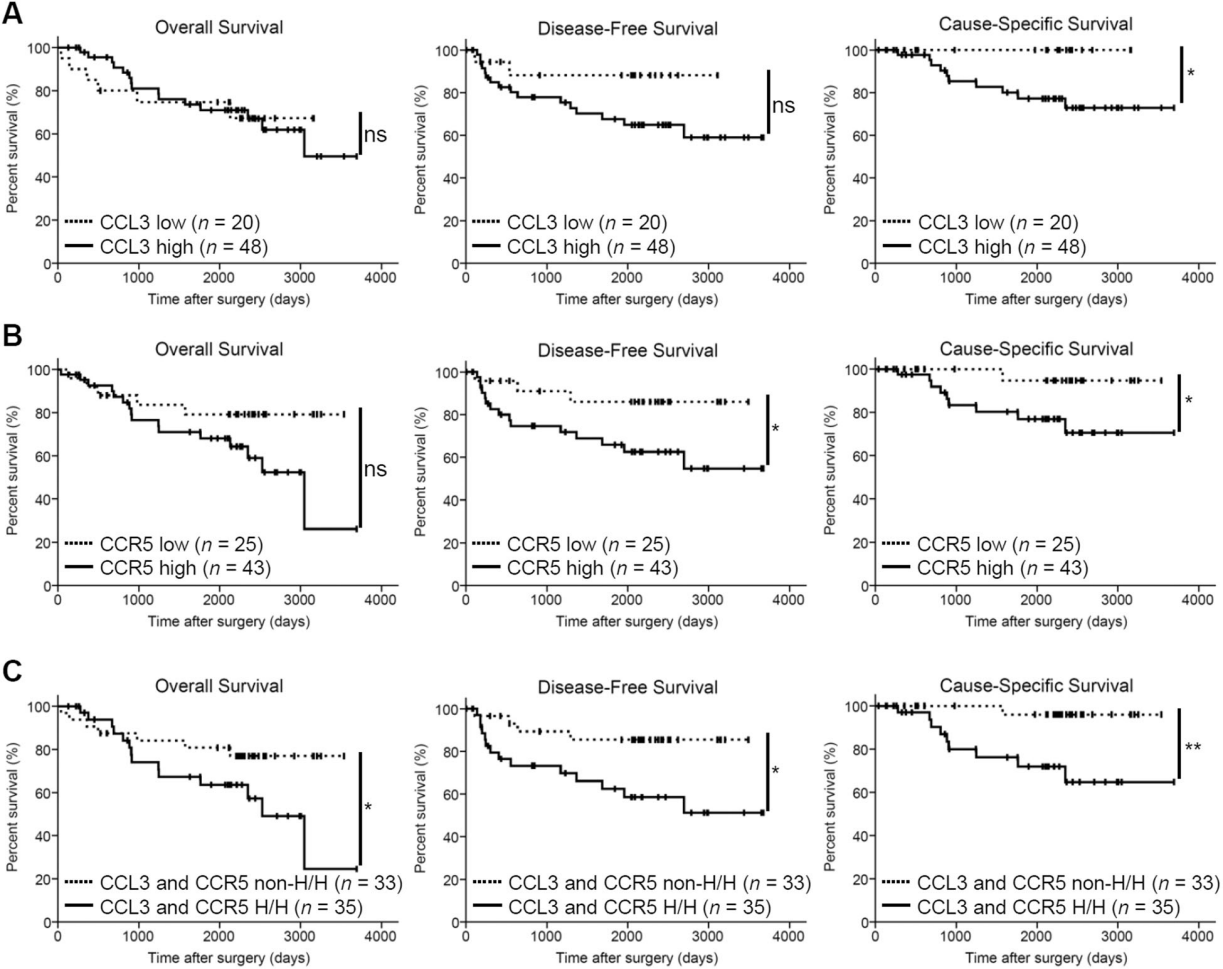
Kaplan–Meier analyses of the overall survival, disease-free survival, and cancer-related survival of ESCC patients. The patients were divided into low and high CCL3 expression groups (**a**); low and high CCR5 expression groups (**b**); high expression of both CCL3 and CCR5 (H/H) and all the other patients (non-H/H) groups (**c**). The data were analyzed by log-rank test (*p < 0.05; **p < 0.01; ns not significant).

A high expression of CCL3 was significantly correlated with the patients’ cause-specific survival (*p* = 0.041) (Fig. 7a), but not with the overall survival or disease-free survival (*p* = 0.951 and 0.101, respectively). In contrast, a high expression of CCR5 was significantly correlated with disease-free survival and cause-specific survival (*p* = 0.043 and 0.047, respectively), but not with overall survival (*p* = 0.089) (Fig. 7b). A high expression of both CCL3 and CCR5 was significantly correlated with not only disease-free survival and cause-specific survival (*p* = 0.018 and 0.007, respectively), but also with the overall survival (*p* = 0.067) (Fig. 7c). A significant independent impact of a high expression of both CCL3 and CCR5 on the disease-free survival rate in ESCC was not detected by the multivariate analysis (*p* = 0.071) (Table 2).

**Table 2:** Univariate and multivariate Cox regression analysis of prognostic factors for disease-free survival.

	*n*	Univariate analysis			Multivariate analysis	
		Mean survival (years)	HR (95% CI)	*p* value	HR (95% CI)	*p* value
Age						
<65	32	7.07	0.654	0.375		
≥65	36	7.91	(0.252–1.691)			
Sex						
Female	14	8.65	2.417	0.189		
Male	54	7.28	(0.555–10.529)			
Histological grade^a^						
IS + WD	15	5.81	1.560	0.485		
MD + PD	53	7.42	(0.448–5.431)			
Depth of invasion^b^						
Tis + T1	48	9.09	12.450	<0.001***	8.580	0.005**
T2 + T3	20	3.59	(4.373–35.450)		(1.933–38.074)	
Lymphatic invasion						
Negative	37	8.91	4.961	0.003**	0.757	0.731
Positive	31	5.84	(1.745–14.106)		(0.155–3.698)	
Vascular invasion						
Negative	43	8.33	2.706	0.038*	1.685	0.366
Positive	25	4.84	(1.059–6.917)		(0.544–5.223)	
Lymph node metastasis						
Negative	43	9.28	8.375	<0.001***	3.388	0.064
Positive	25	4.42	(2.745–25.552)		(0.932–12.322)	
Pathological stage^b^						
0 + I	38	9.47	9.544	<0.001**		
II + III + IV	30	4.69	(2.749–33.133)			
CCL3 expression						
Low	20	7.62	3.206	0.121		
High	48	7.10	(0.736–13.969)			
CCR5 expression						
Low	25	8.50	3.337	0.057		
High	43	6.82	(0.965–11.539)			
CCL3 and CCR5 expression						
Non-H/H	33	8.44	3.534	0.026*	3.136	0.071
H/H	35	6.53	(1.160–10.765)		(0.906–10.856)	
CD68^+^ cells^c^						
Low	35	8.78	3.638	0.009**		
High	33	6.05	(1.293–10.233)			
CD163^+^ cells^c^						
Low	34	8.54	2.591	0.058		
High	34	6.62	(0.969–6.924)			
CD204^+^ cells^c^						
Low	34	8.96	4.755	0.006**	1.176	0.799
High	34	6.04	(1.564–14.461)		(0.337–4.112)	
Microvascular density^d^						
Low	34	7.35	0.832	0.698		
High	34	7.86	(0.328–2.111)			

Data were analyzed by Cox regression analysis. Data were analyzed by *χ*^2^-test.

*p  <  0.05, **p  <  0.01, ***p  <  0.001.

^a^According to the 11th edition of Japanese Classification of Esophageal Cancer: *IS* squamous cell carcinoma (SCC) in situ/high grade intraepithelial neoplasia, *WD* well differentiated SCC, *MD* moderately differentiated SCC, *PD* poorly differentiated SCC [44, 45].

^b^According to the 8th edition of the TNM classification: *Tis* carcinoma in situ/high grade dysplasia, *T1* tumor invades lamina propria, muscularis mucosae, or submucosa, *T2* tumor invades muscularis propria, *T3* tumor invades adventitia [46].

^c^The patients were divided into low and high groups based on the median number of infiltrating CD68^+^, CD163^+^, or CD204^+^ macrophage in tumor cell nests and the tumor stroma [12].

^d^The patients were divided into low and high groups based on the median microvascular density [12].

## Discussion

CCL3 is involved in the progression of various malignancies via CCR1 and/or CCR5 as mentioned above. In most of these above-cited studies, it was described that CCL3 was expressed in neoplastic cells [17, 18, 20, 21, 23–27]. CCL3 expression in nonneoplastic stromal and/or inflammatory cells in the tumor microenvironment was also described in some of those studies [17, 20–22, 24–27]. However, only a few reports investigated the significance of CCL3 derived from inflammatory cells in a tumor microenvironment: CCL3 derived from basophils contributes to the maintenance of leukemia-initiating cells in chronic myeloid leukemia [25], and CCL3 derived from macrophages and granulocytes contributes to the accumulation of CCR5^+^ fibroblasts in murine colitis-associated cancer [22]. In the present study, CCL3 expression was detected in both neoplastic cells and TAMs in vitro as well as in human ESCC tissues. The present study is the first to focus on the role of CCL3 derived from both TAMs and neoplastic cells in the progression of human ESCC. However, the secretion level of CCL3 from TAM-like macrophages was much higher than those from ESCC cell lines. These observations suggest that TAMs are one of the most important sources of CCL3 in human ESCC microenvironment. However, it still remains unknown how conditioned media of ESCC cell lines induce the CCL3 expression in TAM-like macrophages. It has been reported that HIV infection, lipopolysaccharide, and IL-1β induce CCL3 secretion from macrophages [16]. Chen et al. reported that IL-1β was expressed in human ESCC cell [47]. IL-1β was also expressed in M2 TAMs in human ESCC [13, 48]. We suggest that IL-1β derived from ESCC cells and/or TAMs may induce CCL3 secretion from TAMs; however the role of IL-1β against TAMs in ESCC was not well understood.

In the present study, three ESCC cell lines express CCR5 much higher (6.1–9.5-folds) than Het-1A cells, a normal human esophageal squamous epithelial cell line. In contrast, CCR1 expression levels in ESCC cell lines against Het-1A cells were relatively much lower (1.3–1.4-folds) than the CCR5 expression levels. Thus, we focused on the roles of CCL3–CCR5 axis in ESCC. The results of the present study suggested that PI3K/Akt and MEK/ERK pathways were activated downstream of the CCL3–CCR5 axis in ESCC cells. It is well known that Akt and ERK are phosphorylated downstream of the CCL3–CCR5 axis [19, 28, 49]. In addition, there are two sub-pathways in PI3K/Akt, PDK1, and mTORC2. PDK1 phosphorylates Akt on Thr308, whereas mTORC2 phosphorylates Akt on Ser473 [50–52]. In all three ESCC cell lines, rhCCL3 treatment significantly upregulated both p-Akt (Ser473) and p-Akt (Thr308) via CCR5. These observations suggest that the CCL3–CCR5 axis activates Akt via both sub-pathways, PDK1 and mTORC2, in ESCC cells. However, it remains the problem of whether PDK1 or mTORC2 is predominantly activated in each ESCC cell line.

PI3K/Akt and MEK/ERK pathways are activated by many cellular stimuli, and these pathways play key roles in cell regulation including cell growth, survival, migration, invasion, stemness, and metabolism, and they contribute to tumor progression [53, 54]. We observed herein that a CCL3–CCR5 axis promoted the migration and invasion of the three ESCC cell lines via PI3K/Akt and MEK/ERK pathways. Past studies revealed that PI3K/Akt and/or MEK/ERK pathways were also involved in the migration and invasion of ESCC cells in vitro [13–15, 55–57]. CCL3–CCR5 axis-promoted migration and invasion were also reported in oral squamous cell carcinoma [27] and chondrosarcoma [21]. Our present findings are consistent with those of past studies of the CCL3–CCR5 axis and the PI3K/Akt and/or MEK/ERK pathway-derived migration and invasion of neoplastic cells. In the present assay, treatment with Maraviroc or the neutralizing antibody against CCL3 suppressed significantly cell migration and invasion when the ESCC cell lines were co-cultured with TAMs, and suppressed them slightly, but not significantly, when ESCC cell lines were mono-cultured without rhCCL3 treatment. We thus speculate that the CCL3–CCR5 axis plays key role in the progression of ESCC when neoplastic cells are interacting with TAMs. The quantity and the roles that the ESCC cell-derived CCL3 plays in their microenvironment may be limited.

MMP-2 and MMP-9 remodel extracellular matrix and contribute to cell invasion, and VEGF-A promotes angiogenesis in various malignancies [6]. In ESCC, MMP-2 and MMP-9 contribute to tumor invasion and metastasis [58, 59], and VEGFs including VEGF-A contribute to angiogenesis and are associated with high microvascular density and poor patient prognosis [60–62]. MMP-2 is involved in CCL3-dependent cell invasion in oral squamous cell carcinoma [27] and chondrosarcoma [21], whereas MMP-9 is involved in CCL3-dependent cell invasion in hepatocellular carcinoma [17] and oral squamous cell carcinoma [27]. VEGF-A is involved in CCL3-dependent angiogenesis in osteosarcoma [23] and is also upregulated downstream of the CCL3–CCR5 axis in oral squamous cell carcinoma [27]. Activating the PI3K/Akt and MEK/ERK pathways induces the expressions of MMP-2, MMP-9, and VEGF-A in various malignancies including ESCC [62–65]. Our present results demonstrated that treatment with rhCCL3 upregulated the *MMP2* mRNA expression in three ESCC cell lines universally, and the *VEGFA* mRNA expression in two ESCC cell lines. All upregulated expressions of *MMP2* and *VEGFA* after rhCCL3 treatment were suppressed by LY294002 and PD98059. We thus speculate that in ESCC, MMP-2 is involved in the CCL3–CCR5 axis-derived cell invasion via PI3K/Akt and MEK/ERK pathways, and VEGF-A contributes to angiogenesis via the CCL3–CCR5 axis and PI3K/Akt and MEK/ERK pathways.

In contrast, rhCCL3 treatment upregulated the *MMP9* mRNA expression only in TE-15 cells, and paradoxically, the upregulated expression of *MMP9* by rhCCL3 was further upregulated by LY294002 and PD98059. CCL3-dependent *MMP9* upregulation in ESCC cells may be not universal, and may occur via other signaling pathways, such as p38 MAPK, AMPK, or NF-κB, which were also reported to be activated downstream of CCL3–CCR5 axis [21, 49]. The paradoxical *MMP9* upregulation by LY294002 and PD98059 with rhCCL3 may be caused by the compensatory upregulation of other pathways due to Akt and ERK inhibition.

However, there still remains the problem why the *MMP9* and *VEGFA* upregulations differ between each cell line. No association between upregulations and poor differentiation was evident. Past studies showed that there was no association between MMP9 or VEGFA expression, and the histological grade in ESCC [58, 60]. Our clinical data showed that CCL3 and/or CCR5 expression did not associate with histological grade. Differences between the *MMP9* and *VEGFA* upregulations do not seem to rely on the differentiation of ESCC cell lines. These differences may occur by the different genetic background, however the genetics of TE series ESCC cell lines were not revealed in full [43].

Herein, we detected immunoreactivities for both CCL3 and CCR5 in the tumor nests of human ESCC tissues. These observations are consistent with those of immunohistochemical and immunofluorescent examinations of CCL3 and CCR5 in human or murine cancer tissues [17, 20, 21, 23, 26, 27, 32, 34–36]. Our examination of double IF showed that CCL3 was expressed in both neoplastic cells and TAMs in the tumor nest of human ESCC tissue. In the present immunohistochemical assay, it was difficult to distinguish the CCL3 expression in neoplastic cells or nonneoplastic inflammatory cells, because of its diffuse and uniform staining pattern. However, the secretion level of CCL3 in the three ESCC cell lines was much lower than that in PBMo-derived TAM-like macrophages. This discrepancy suggested that the results of the past and present immunohistochemical studies of CCL3 might not adequately reflect the CCL3 secretion levels in tissue.

We observed that high CCL3 expression in the human ESCC tissue samples was significantly associated with poor cause-specific survival but not with disease-free survival, overall survival, or any of the clinicopathological factors. In contrast, high CCR5 expression in the human ESCC tissue samples was significantly associated with poorer disease-free and cause-specific survival, deeper invasion, vascular invasion, higher pathological stage, higher numbers of infiltrating CD204^+^ TAMs, and higher microvascular density. A high expression of CCR5 was more closely associated with poor prognosis and clinicopathological factors compared with a high expression of CCL3. This may be explained by the following: (1) These are other CCR5 ligands, e.g., CCL4, CCL5, CCL8, and CCL3L1 [29, 30]. We have demonstrated that CCL4, CCL5, and CCL8 are also upregulated in TAM8, TAM9, and TAM15 cells [13]. The CCL4–CCR5, CCL5–CCR5, and CCL8–CCR5 axes appear to be involved in the progression of ESCC. (2) As mentioned above, we suggest that CCL3 immunohistochemistry may not preciously reflect the CCL3 secretion levels in tissue, especially in neoplastic cells. The clinicopathological association between high CCR5 expression and deeper invasion supports the findings of promoted cell invasion and upregulated *MMP2* mRNA expression in the three ESCC cell lines by rhCCL3 treatment. In addition, the clinicopathological association between high CCR5 expression and presence of vascular invasion or higher microvascular density supports our observation of upregulated *VEGFA* mRNA expression in the ESCC cell lines following the rhCCL3 treatment.

There are a few reports which have found that high levels of CCL3 and/or CCR5 expressions in human cancer tissue were associated with poor prognosis. In oral squamous cell carcinoma, the mean survival rate for patients with high numbers of CCL3-positive cells in the tumor parenchyma was shorter than that of the patients with low numbers of CCL3-positive cells, although not significantly [26]. A high plasma level of CCL3 was associated with poor survival rate in chronic lymphocytic leukemia [66] and diffuse-large B-cell lymphoma [67]. CCR5 was reported to be associated with poor overall survival in breast cancer [34], poor relapse-free survival in prostate cancer [68], high histological grade in pancreatic cancer [35], and perineural invasion in salivary adenoid cystic carcinoma [36]. The present study is the first report which revealed that the expression of both CCL3 and CCR5 in human cancer tissue is associated with poor patient prognosis.

Moreover, high expression of both CCL3 and CCR5 in the human ESCC tissue was significantly associated with poor overall, disease-free and cause-specific survivals. However, it could not show a significant independent impact on the disease-free survival rate, the *p* value (0.071) was very close to the significance cut-off (0.05). A CCL3–CCR5 axis may therefore play a key role in the tumor progression, and it could be the target of new therapies against ESCC. In this study, the CCR5 antagonist, Maraviroc, inhibited the CCL3–CCR5 axis-dependent ESCC migration and invasion. Maraviroc is used clinically as an anti-HIV agent and has shown few side effects, and it was reported to reduce lung metastasis and inhibit the tumor growth of breast cancer in mice [31, 34, 40], inhibit murine xenograft growth of classical Hodgkin lymphoma [37], and inhibit the CCL5-dependent migration and invasion of ESCC cell lines [38]. Many other types of CCR5 antagonists and inhibitors such as Vicriviroc, TAK-779, Met-CCL5 (Met-RANTES), OTR4120, OTR4131, anibamine, and DT-13 were also reported to inhibit the cell migration, invasion and/or metastasis of various malignancies including breast cancer, gastric cancer, pancreatic cancer and hepatocellular carcinoma in preclinical studies [34, 69].

**Fig. 8:**
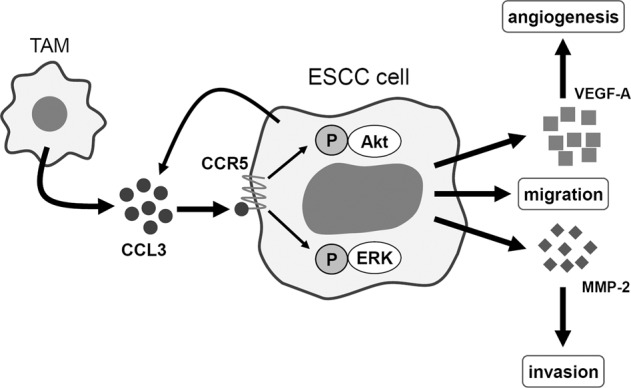
Schematic model of the CCL3–CCR5 axis in esophageal squamous cell carcinoma. CCL3 is derived from both TAMs and cancer cells and is bound to CCR5 on cancer cells. CCL3–CCR5 interaction contributes to the progression of ESCC by activating Akt and ERK signaling pathways and by promoting the migration and invasion of cancer cells and angiogenesis.

In conclusion, TAMs and ESCC cells express and secrete CCL3. CCL3 contributes to tumor progression and poor prognosis in ESCC patients by binding CCR5 on ESCC cells, activating PI3K/Akt and MEK/ERK pathways, upregulating MMP-2 and VEGF-A expression, and promoting cell migration, invasion, and angiogenesis (Fig. 8). CCR5 antagonists, which are usually used for treating HIV, could also be effective agents for the control of ESCC progression.

## Supplementary information

Supplemental Figures_S1_S2_S3_S4_S5_S6_S7
