# Supplementary material for: CCL3–CCR5 axis contributes to progression of esophageal squamous cell carcinoma by promoting cell migration and invasion via Akt and ERK pathways
Source: Lab Invest. 2020 May 26;100(9):1140–57. doi: 10.1038/s41374-020-0441-4 (PMC7438203; doi:10.1038/s41374-020-0441-4)
Supplement: Supplementary file 1 — Supplemental Figures_S1_S2_S3_S4_S5_S6_S7 [file 41374_2020_441_MOESM1_ESM.pdf]

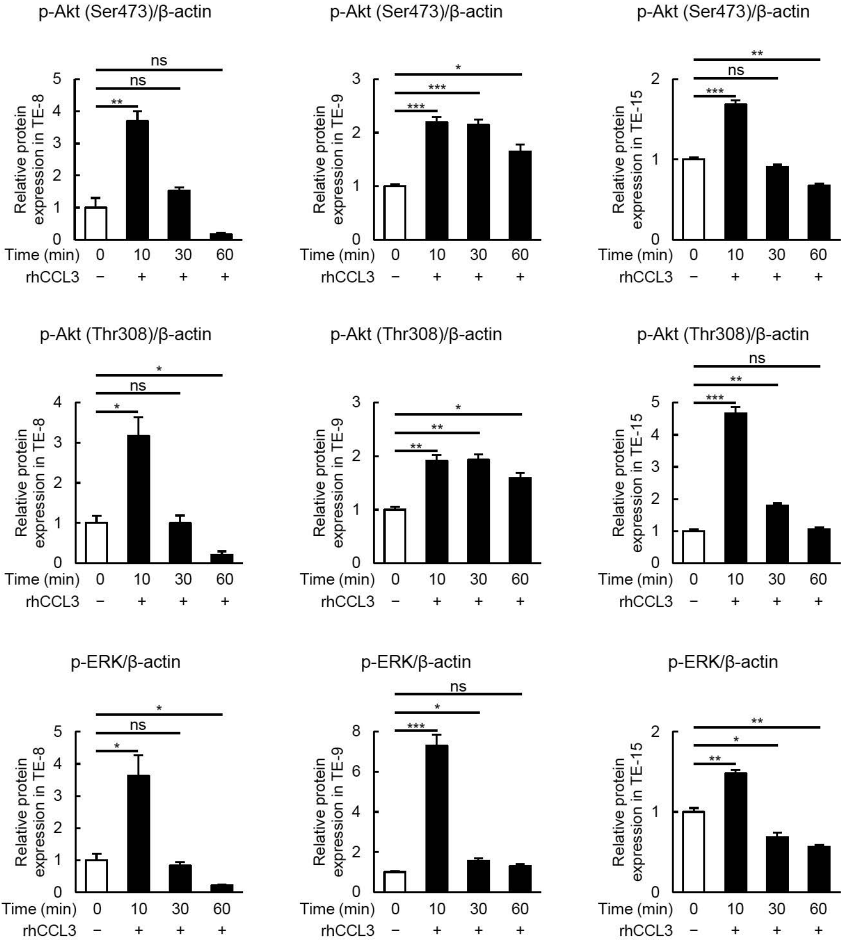

Suppl. Fig. S1. Densitometric analyses of the western blotting bands shown in Fig. 3A. Results are mean ± SEM (n = 3; *p < 0.05; **p < 0.01; ***p < 0.001; ns, not significant).

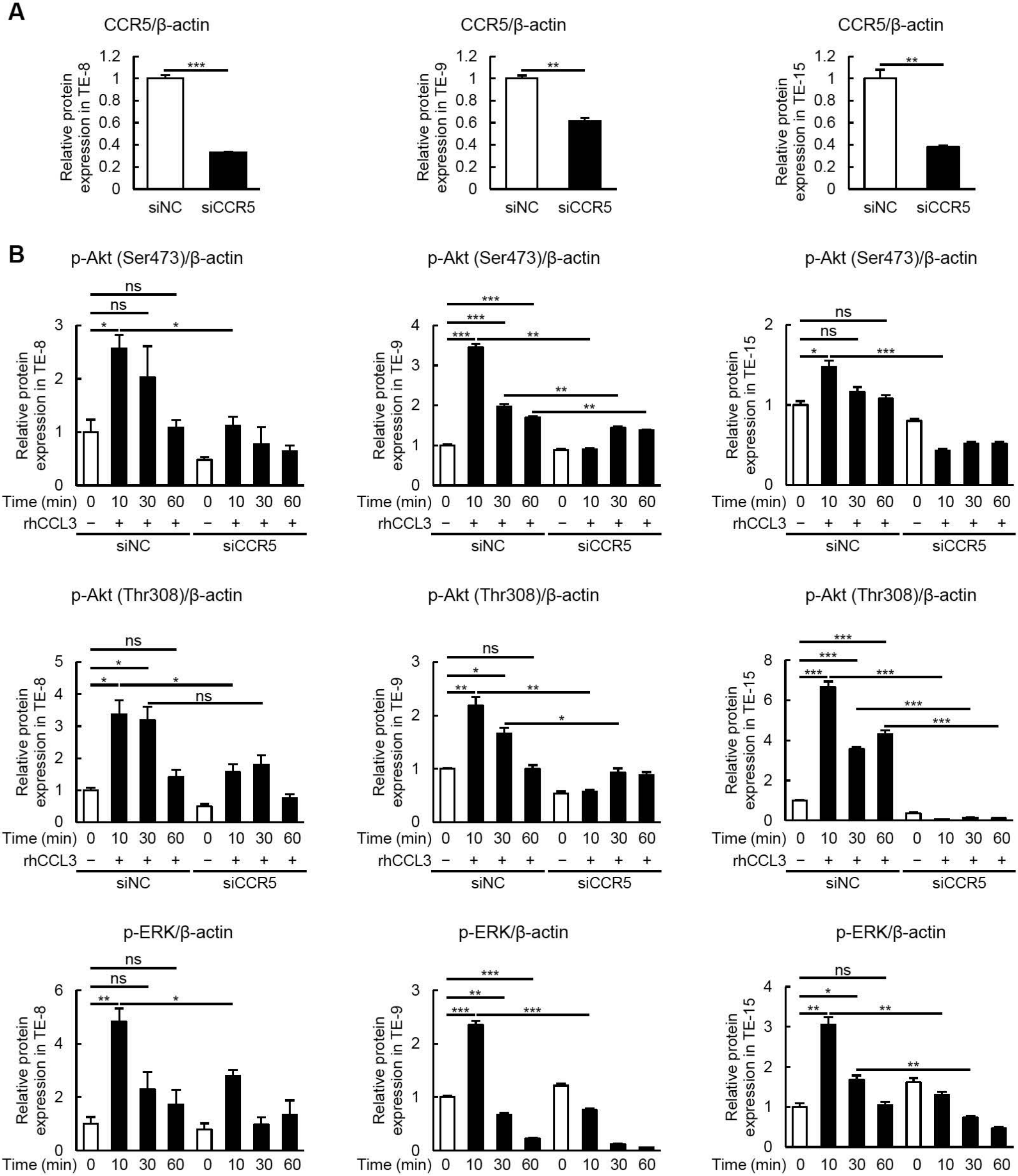

Suppl. Fig. S2. Densitometric analyses of the western blotting bands shown in Fig. 3C (**A**) and 3D (**B**). Results are mean ± SEM (n = 3; *p < 0.05; **p < 0.01; ***p < 0.001; ns, not significant).

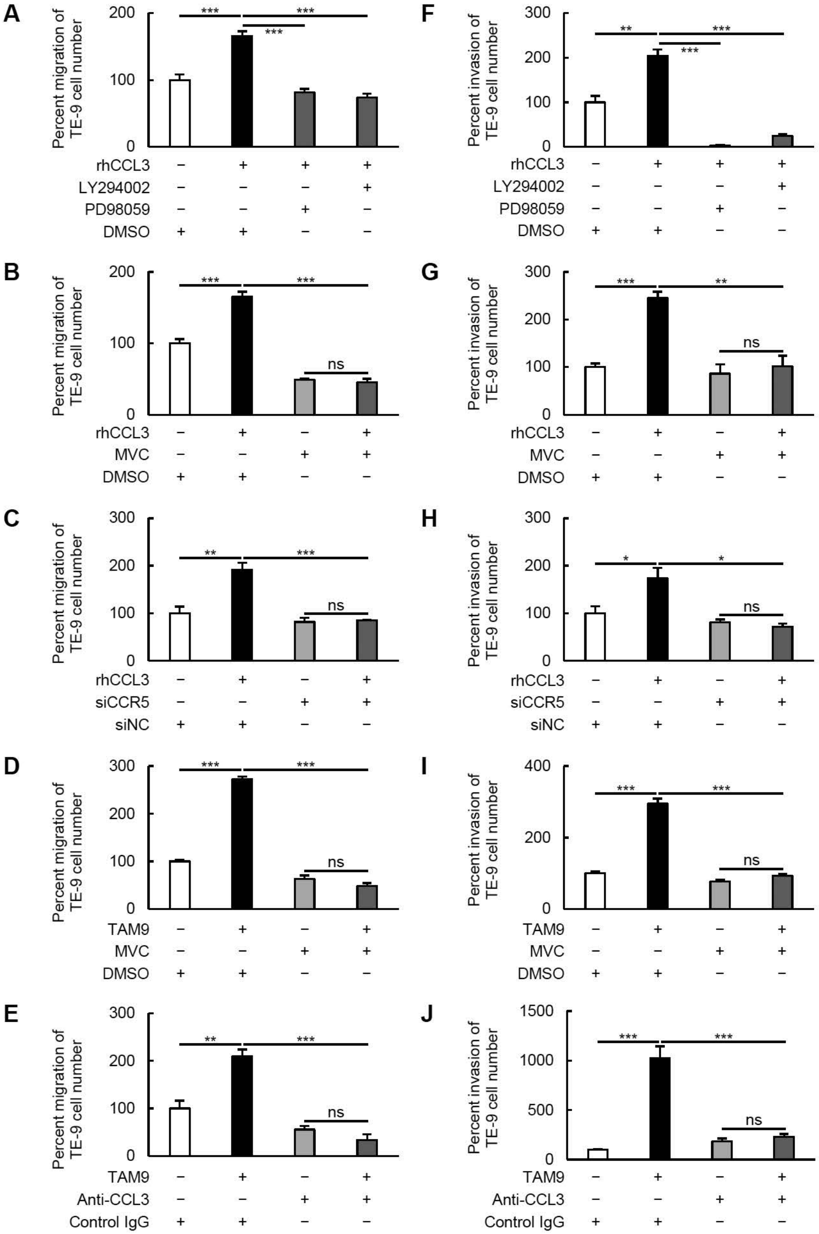

Suppl. Fig. S3. Transwell migration and invasion assays of TE-9 cells. **A–E:** For the migration assay, 1×10^5 TE-9 cells were plated on the transwell in RPMI1640 medium containing 0.1% FBS. Then 100 ng/ml rhCCL3 (**A–C**) or 1×10^5 TAM9 (**D, E**) was added in the lower chamber. The cell inserts were set on 24-well plates for 24 h. The migrated and invaded cells on the underside of the membrane were stained and counted. Results are mean ± SEM (n = 3; *p < 0.05; **p < 0.01; ***p < 0.001; ns, not significant). In each assay, the percent migration or invasion was calculated by dividing the number of TE-9 cells by that in the negative control. **F–J:** For the invasion assay, TE-9 cells were plated on the transwell in the same condition, and rhCCL3 (**F–H**) or TAM9 (**I, J**) was added in the lower chamber. The cell inserts were incubated for 48 h. **A, F:** 20 μM LY294002 and PD98059 were added on the upper chamber, and 0.2 μl/ml DMSO was added as a negative control. **B, D, G, I:** 20 μg/ml Maraviroc (MVC) was added on the upper chamber, and 0.2 μl/ml DMSO was added as a negative control. **C, H:** 2×10^5 TE-9 cells transfected with 20 nM siCCR5 or siNC were plated. **E, J:** 400 ng/ml CCL3 neutralizing antibody (anti-CCL3) was added on the lower chamber, and 400 ng/ml control IgG was added as a negative control.

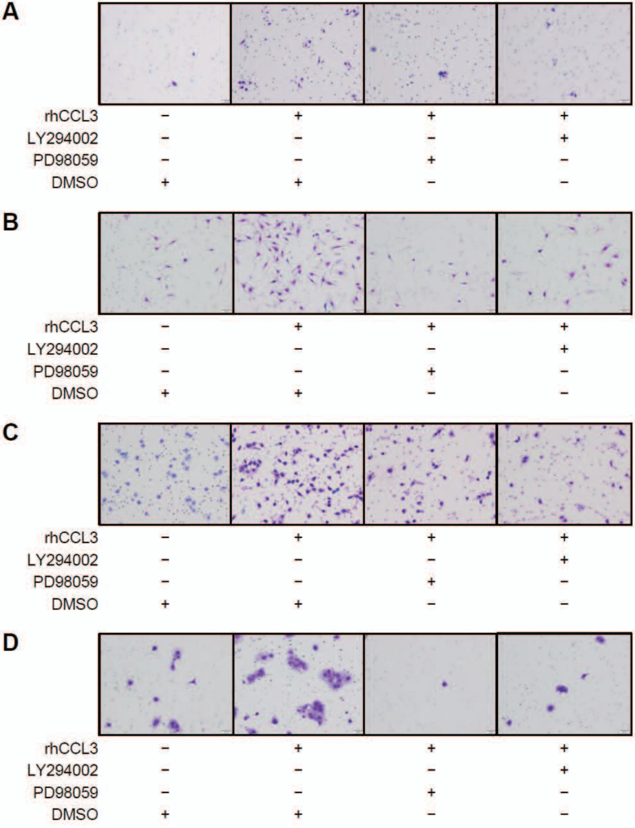

Suppl. Fig. S4. Representative images of the results of transwell migration and invasion assays. **A, C:** Representative images of the results of transwell migration assays. TE-8 (**A**) or TE-9 (**C**) cells were plated on the transwell. rhCCL3 was added in the lower chamber. LY294002 and PD98059 were added on the upper chamber, and DMSO was added as a negative control. **B, D:** Representative images of the results of transwell invasion assays. TE-8 (**B**) or TE-9 (**D**) cells were plated on the transwell. rhCCL3 was added in the lower chamber, and LY294002, PD98059 and DMSO were added on the upper chamber.

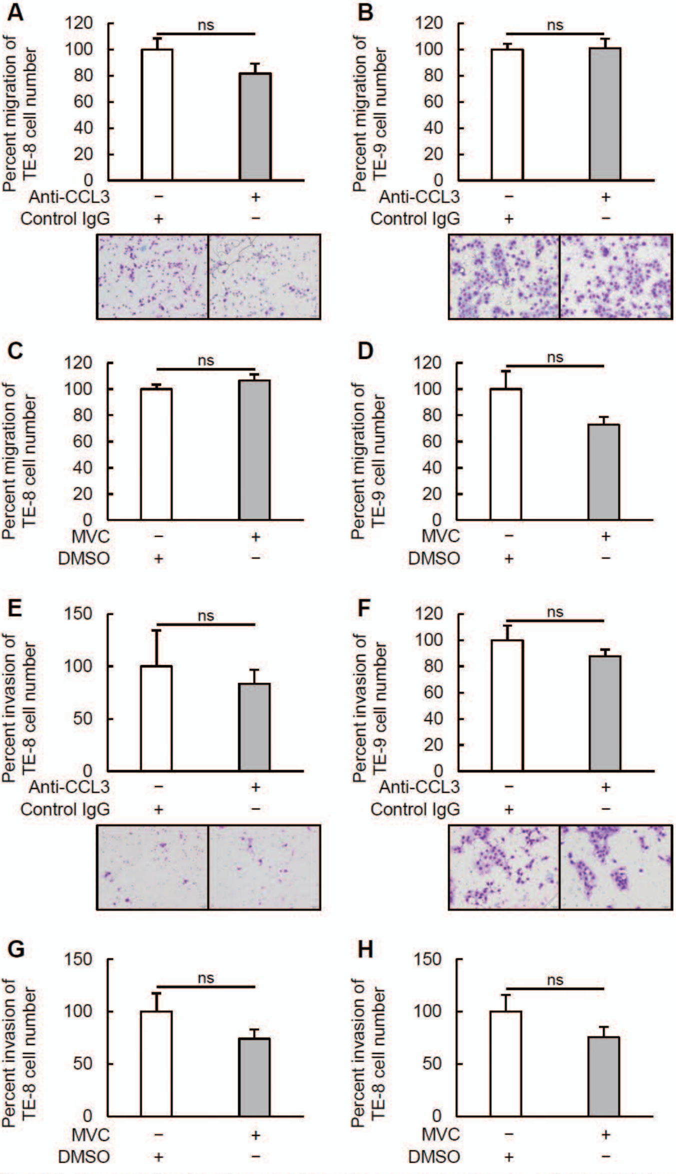

Suppl. Fig. S5. Transwell migration and invasion assays of mono-cultured TE-8 and TE-9 cells. 2×10^5 TE-8 (**A, C, E, G**) or 1×10^5 TE-9 (**B, D, F, H**) cells were plated on the upper chamber in RPMI1640 medium containing 0.1% FBS. RPMI1640 medium containing 1% FBS was added on the lower chamber. **A–D:** For the migration assay, the cell inserts were set for 24 h. **E–H:** For the invasion assay, the cell inserts were set for 48 h. **A, B, E, F:** 400 ng/ml CCL3 neutralizing antibody (anti-CCL3) was added on the upper chamber, and 400 ng/ml control IgG was used as a negative control. **C, D, G, H:** 20 μg/ml Maraviroc (MVC) was added on the upper chamber, and 0.2 μl/ml DMSO was added as a negative control. Results are mean ± SEM (*n* = 3; ns, not significant). **A, B, E, F:** The Representative image was shown below each graph. In each assays, the percent migration or invasion was calculated by dividing the number of TE-8 or TE-9 cells by that in the negative control.

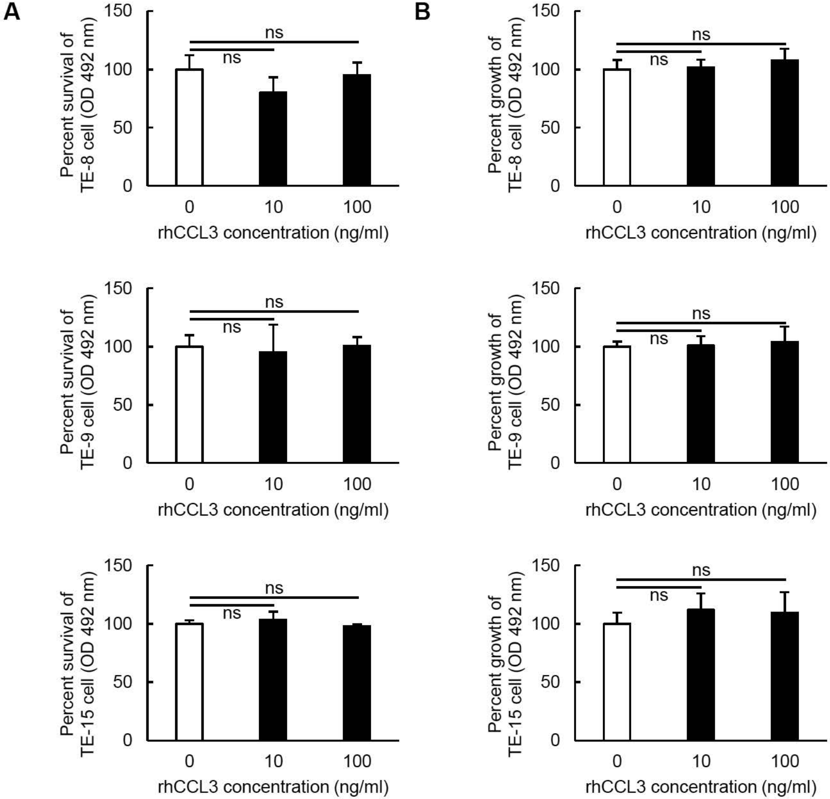

Suppl. Fig. S6. Cell survival and growth assays of TE-8, TE-9, and TE-15 cells. **A:** For the cell survival assay, 1×10^4 ESCC cell lines were plated in serum-free medium and incubated for 72 h. An MTS assay was performed and the optical density (OD) at 492 nm was determined. Treatment with 10 or 100 ng/ml rhCCL3 did not significantly improve the OD in TE-8, TE-9, or TE-15 cells (*n* = 4; ns, not significant). **B:** For the cell growth assay, 5×10^3 of each ESCC cell lines were plated in medium supplemented with 0.1% FBS and incubated for 72 h. The MTS assay was performed, and the OD at 492 nm was determined. Treatment with 10 or 100 ng/ml rhCCL3 did not significantly improve the in TE-8, TE-9, or TE-15 cells (*n* = 4; ns, not significant).

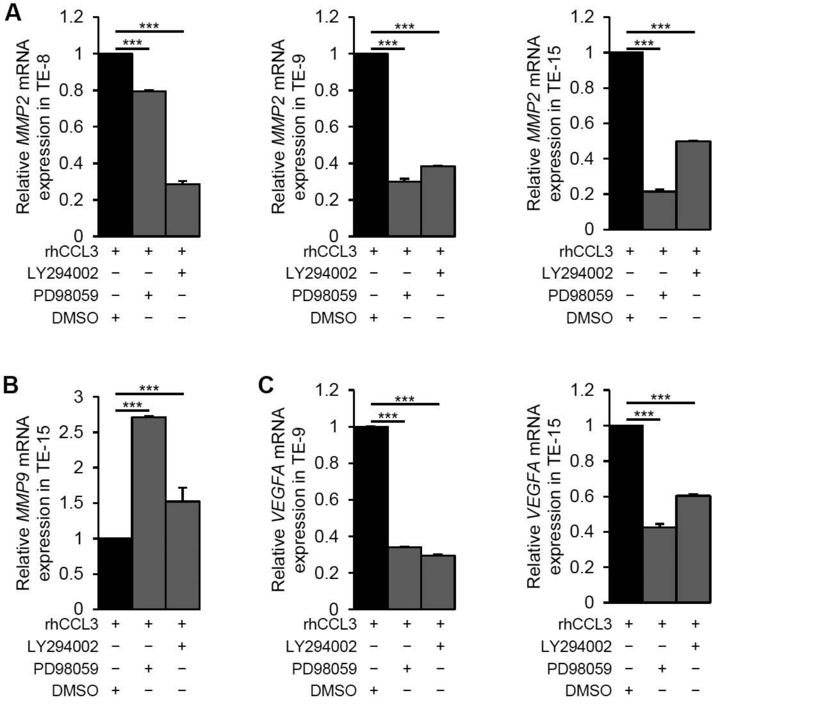

Suppl. Fig. S7. The expressions of *MMP2*, *MMP9*, and *VEGFA* in 5×10^5 TE-8, TE-9, and TE-15 cells under serum-free conditions treated with 100 ng/ml rhCCL3 and 20μM LY294002 or PD98059. TE-8 and TE-15 cells were treated with rhCCL3 and LY294002 or PD98059 for first 24 h, then the cells were treated with only rhCCL3 for later 24 h. TE-9 cells were treated with rhCCL3 and LY294002 or PD98059 for 48 h. **A:** *MMP2* (TE-8, TE-9, TE-15), **B:** *MMP9* (TE-15), and **C:** *VEGFA* (TE-8, TE-9) mRNA expression levels were determined by quantitative RT-PCR and normalized to *GAPDH* expression. Data are mean ± SEM (n = 3, ***$p < 0.001$).
